# Early and Subsequent Epidemic Characteristics of COVID-19 and Their Impact on the Epidemic Size in Ethiopia

**DOI:** 10.3389/fpubh.2022.834592

**Published:** 2022-05-11

**Authors:** Abebe Feyissa Amhare, Yusha Tao, Rui Li, Lei Zhang

**Affiliations:** ^1^Department of Epidemiology and Health Statistics, School of Public Health, Xi'an Jiaotong University Health Science Center, Xi'an, China; ^2^Department of Public Health, College of Health Science, Salale University, Fitche, Ethiopia; ^3^China-Australia Joint Research Center for Infectious Diseases, School of Public Health, Xi'an Jiaotong University Health Science Center, Xi'an, China; ^4^Melbourne Sexual Health Centre, Alfred Health, Melbourne, VIC, Australia; ^5^Faculty of Medicine, Nursing and Health Sciences, Central Clinical School, Monash University, Melbourne, VIC, Australia

**Keywords:** COVID-19, epidemic size, early epidemic indicators, early characteristics of COVID-19, Ethiopia

## Abstract

In Ethiopia, multiple waves of the COVID-19 epidemic have been observed. So far, no studies have investigated the characteristics of the waves of epidemic waves in the country. Identifying the epidemic trend in Ethiopia will inform future prevention and control of COVID-19. This study aims to identify the early indicators and the characteristics of multiple waves of the COVID-19 epidemics and their impact on the overall epidemic size in Ethiopia. We employed the Jointpoint software to identify key epidemic characteristics in the early phase of the COVID-19 epidemic and a simple logistic growth model to identify epidemic characteristics of its subsequent waves. Among the first 100 reported cases in Ethiopia, we identified a slow-growing phase (0.37 [CI: 0.10–0.78] cases/day), which was followed by a fast-growing phase (1.18 [0.50–2.00] cases/day). The average turning point from slow to fast-growing phase was at 18 days after first reported. We identified two subsequent waves of COVID-19 in Ethiopia during 03/2020-04/2021. We estimated the number of COVID-19 cases that occurred during the second wave (157,064 cases) was >2 times more than the first (60,016 cases). The second wave's duration was longer than the first (116 vs. 96 days). As of April 30th, 2021, the overall epidemic size in Ethiopia was 794/100,000, ranging from 1,669/100,000 in the Harari region to 40/100,000 in the Somali region. The epidemic size was significantly and positively correlated with the day of the phase turning point (r = 0.750, *P* = 0.008), the estimated number of cases in wave one (r = 0.854, *P* < 0.001), and wave two (r = 0.880, *P* < 0.001). The second wave of COVID-19 in Ethiopia is far greater, and its duration is longer than the first. Early phase turning point and case numbers in the subsequent waves predict its overall epidemic size.

## Introduction

In late December 2019, the Severe Acute Respiratory Syndrome Coronavirus 2 (SARS-COV-2) was first reported in Wuhan City, China (1–3). The disease was later named coronavirus disease COVID-19 by the World Health Organization (WHO) (4, 5). The COVID-19 epidemic has since spread at an alarming rate worldwide. As of September 21^st^, 2021, the total number of confirmed COVID-19 cases exceeded 206 million, and the death toll passed 4.7 million. Countries have implemented orchestrated efforts to confine the COVID-19 epidemic (6–8). Non-pharmaceutical interventions, including social distancing, face mask use and vaccination, have been shown to be effective in slowing the spread of COVID-19 (9–12).

Ethiopia confirmed its first case of COVID-19 on March 13^th^, 2020 (13). Since then, the Ethiopian government has adopted various strategies to prevent the spread of COVID 19. With the increase in the number of new cases, the Ethiopian government declared a five-month national emergency on April 8th, 2020, after the number of confirmed cases reached 55, but allowed economic activities to continue (14). After declaring a national emergency, the government and the Ethiopian Ministry of Health implemented strict public health measures. These measures included closing schools, restricting large gatherings, including religious gatherings in churches and mosques. Although public transportation is highly transmissible channel for COVID-19, it was only partially limited in Ethiopia. A face mask was mandatory in crowded places and public service places. Social distancing and handwashing with soap were the main control measures and were widely broadcast on the media (15). However, the lockdown was not strictly implemented due to the fragility in the country's economy and people's socio-economic conditions. This endemic disrupts the economy and increases the healthcare system's burden (16–19). Economic activities, especially agricultural and industrial activities, were necessary to continue to maintain food security. During the lockdown, the number of new cases reported daily increased dramatically. As of April 30^th^, the total number of confirmed SARS-COV-2 cases passed 257,442 and 3,688 deaths were reported in Ethiopia. Since February 2021, the number of new confirmed cases and death cases have been dramatically increasing (20). Stronger public health measures needed to be in place to prevent the further spread of the virus.

In Ethiopia, multiple waves of the COVID-19 epidemic has been observed. However, no studies investigated epidemic indicators of COVID-19 during the early phase of the epidemic and its subsequent waves in Ethiopia. Identifying the epidemic trend in Ethiopia will help inform future prevention and control of the epidemic. Modeling studies have been widely used to investigate the trend of the COVID-19 epidemic and evaluate relevant interventions (21–27). Previous studies demonstrated that the epidemic's early characteristics are useful in projecting the subsequent epidemics (28, 29). The research aims to identify the epidemic characteristics of COVID-19 in its early stage and multiple subsequent waves and their association with Ethiopia's overall epidemic size.

## Materials and Methods

### Source of Data

We collected publicly available data related to COVID-19, such as daily confirmed cases, cumulative cases, recovery, and deaths cases from 10 regions and two administrative cities in Ethiopia from March 13^th^, 2020, to April 30^th^, 2021.

### Determining the Early Characteristics of the First Wave of COVID-19

**Figure 1:**
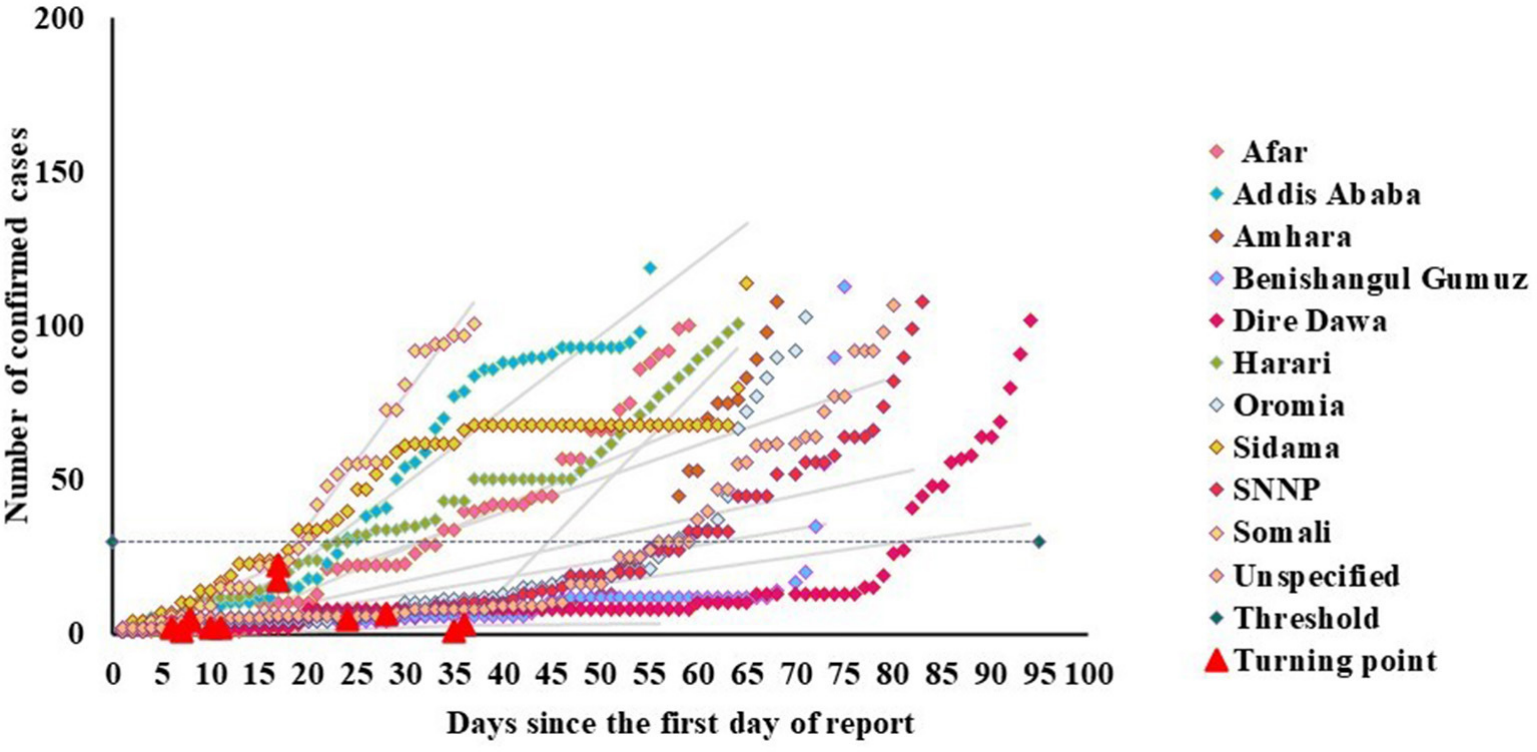
Joinpoint two-phase fitting for Ethiopia regional states, showing the transition point below a threshold of 30 cases.

Early epidemic indicators, such as the turning point time, the number of cases at the turning point, the slow growth phase and the rapid growth phase, the number of days required to increase from 30 to 100 cases, and the case fatality rate (CFR-100) of the first 100 confirmed cases were estimated by using the Joinpoint software (30) based on the first 100 confirmed cases (28). All of turning points occurred below 30 cases (Figure 1). Due to this, we used 30 cases as the threshold to indicate that the epidemic has changed from a slow-growing phase to a rapid-growing phase.

### Determining the Characteristics of Multiple Waves of COVID-19

Based on the cumulative confirmed COVID-19 cases, the epidemic's key characteristics were identified using the bi-logistic growth model (https://logletlab.com) among 10 regions and two administrative cities in Ethiopia. The methods of simple logistic function have been documented in previous studies (31, 32). Like the previous study conducted in Australia (29), we used the logistic growth method to know the current status of COVID-19 in Ethiopia and predict its characteristics for the upcoming months. We model the epidemic patterns by identifying one to two growth waves of the COVID-19 epidemic. By this model, the level at which epidemic saturate (K), the midpoint of each epidemic growth (t_m_), the lengths of time intervals (Δt) required for the epidemic to grow from 10 to 90% of the saturation level in both waves were identified.

### Determining the Overall Epidemic Size of COVID-19

The epidemic size is defined as the total number of confirmed cases as of April 30^th^, 2021, divided by the population size of each region and administrative city and then multiplied by 100,000 individuals.

### Statistically Analysis

Spearman's correlation test was conducted to determine the correlation between epidemic size and bi-logistic parameters. In addition, the correlation between epidemic size and early-stage epidemic indicators was performed. We compared the differences between K_1_ and K_2_ parameters by using nonparametric Mann-Whitney tests.

## Results

### Early Characteristics of the COVID-19 Epidemic

This study demonstrated two-phase linear fits to the first 100 confirmed cases of COVID-19 during the early phase of the epidemic in the ten regions and two administrative cities of Ethiopia. Table 1 illustrates the early-stage epidemic characteristics in Ethiopia. We have identified the slow and fast-growing phases in the early phase of the 100 confirmed cases. The average day that the slow-growing phase turned to the fast-growing phase was 18.09 (12.36–24.82) days. The growth rate in the slow-growing phase was 0.37 (CI: 0.10–0.78) cases/day, whereas, in the fast-growing phase, it was 1.18 (CI: 0.50–2.00) cases/day. This indicated that the fast-growing phase was 0.81 times higher than the slow-growing phase. Based on a previous study, the 30 confirmed cases as a critical threshold where the COVID-19 epidemic started to increase rapidly (28). About 82% of the regional states of Ethiopia transited from slow-growing phase to fast-growing phase at a level below 30 cases, as described in Figure 1. Besides, the average number of days required to increase from 30 to 100 cases was 22 (CI: 14.91–24.64). The average number of cases at the phase transition point in Ethiopia was 11(CI: 4.55–19.08). The average case-fatality rate in the first 100 confirmed cases across all regional states was 1.93 (CI: 0.79–3.05).

**Table 1:** Early indicators in the early-stage of the epidemic in each regional state of Ethiopia.

**Region**	**Number of confirmed cases at the date the 100th cases were reported**	**Number of deaths at the date the 100th cases were reported**	**Time from 30-to-100 cases**	**Case fatality rate in the first 100 confirmed cases**	**Day of the phase turning point**	**Number of cases at turning point**	**Slow growing phase (cases/day)**	**Fast growing phase (cases/day)**	**Epidemic size/100,000**
Addis Ababa	119	4	31	3.36%	36	3	0.01	0.11	4,851
Afar	100	0	25	0.00%	11	1	0.05	1.4	142
Amhara	108	0	11	0.00%	6	2	0.01	0.2	49
Benishangul Gumuz	113	0	4	0.00%	10	2	0.01	0.17	322
Dire Dawa	102	4	12	3.92%	28	8	0.25	0.3	1,056
Harari	101	4	40	3.96%	35	43	0.03	2.63	1,669
Oromia	103	3	12	2.91%	24	5	0.15	0.6	101
Sidama	114	6	46	5.26%	17	24	1.7	2.22	254
SNNP	108	2	22	1.85%	7	5	0.2	0.61	49
Somali	101	0	17	0.00%	17	23	1.5	4.26	40
Unspecified	107	0	23	0.00%	8	5	0.2	0.5	199
Mean (Confidence intervals)	106.9 (103.45–110.63)	2.10 (0.90–3.45)	22 (14.91–24.64)	1.93 (0.79–3.05)	18.09 (12.36–24.82)	11 (4.55–19.08)	0.37 (0.10–0.78)	1.18 (0.50–2.00)	794 (159–1,668)

*SNNP; Southern Nations Nationalities and People. Unspecified: refers to areas where we cannot obtain public data. It is obtained by subtracting the data of 9 regions and 2 cities which have publicly available data from the national data*.

### Characteristics of Subsequent COVID-19 Outbreaks

**Figure 2:**
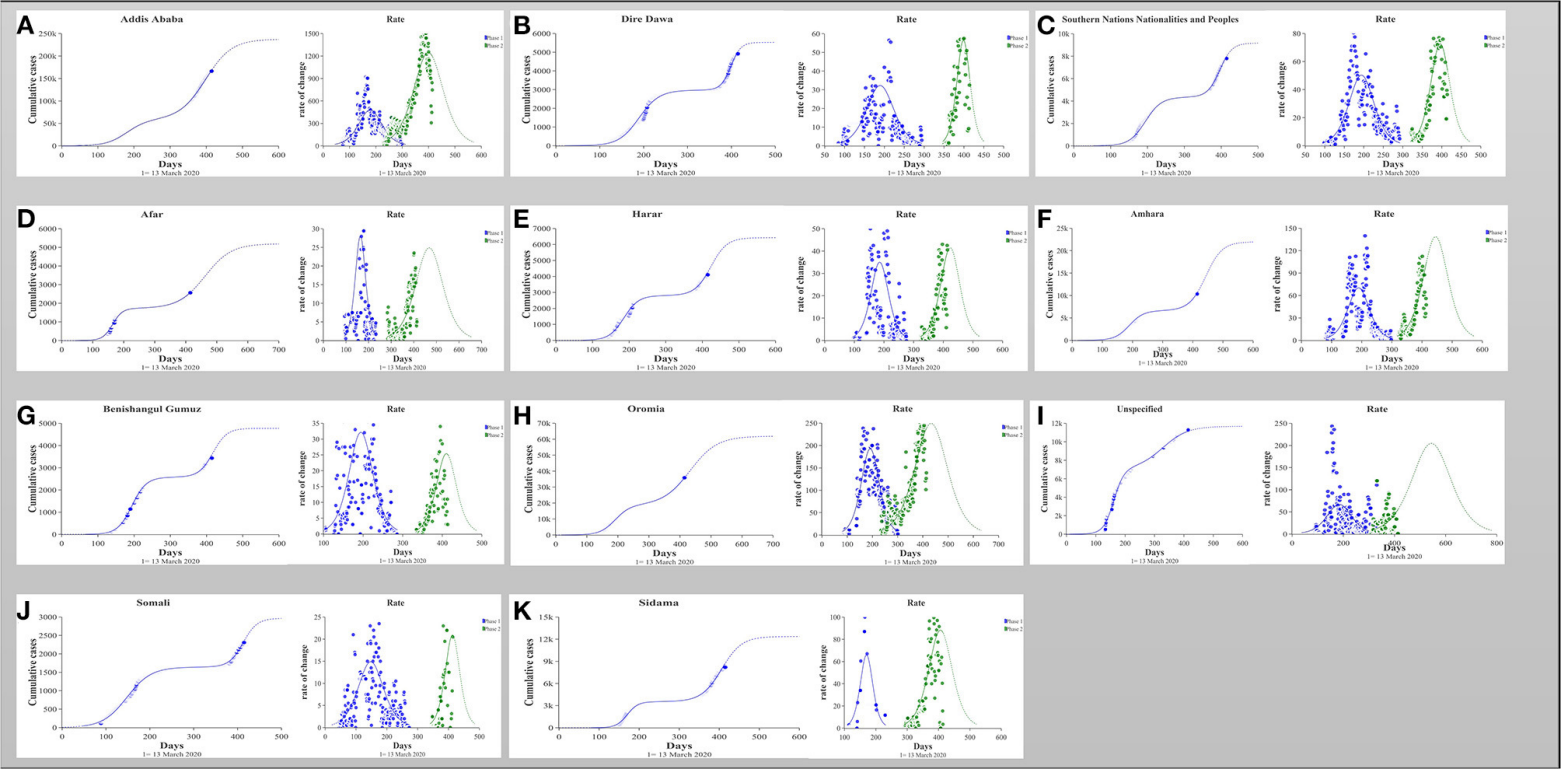
**(A–K)** The number of cumulative cases was calibrated to a simple bi-logistic function, which was used to model biologic patterns with two growth waves. The parameters *K* represent the asymptotic value that bound the function and therefore specify the level at which the cases saturate; *t*_*m*_ represents the midpoint of the epidemic growth and hence the peak of the outbreak; Δ*t* are the lengths of time intervals required for the epidemic to grow from 10 to 90% of the saturation level.

**Figure 3:**
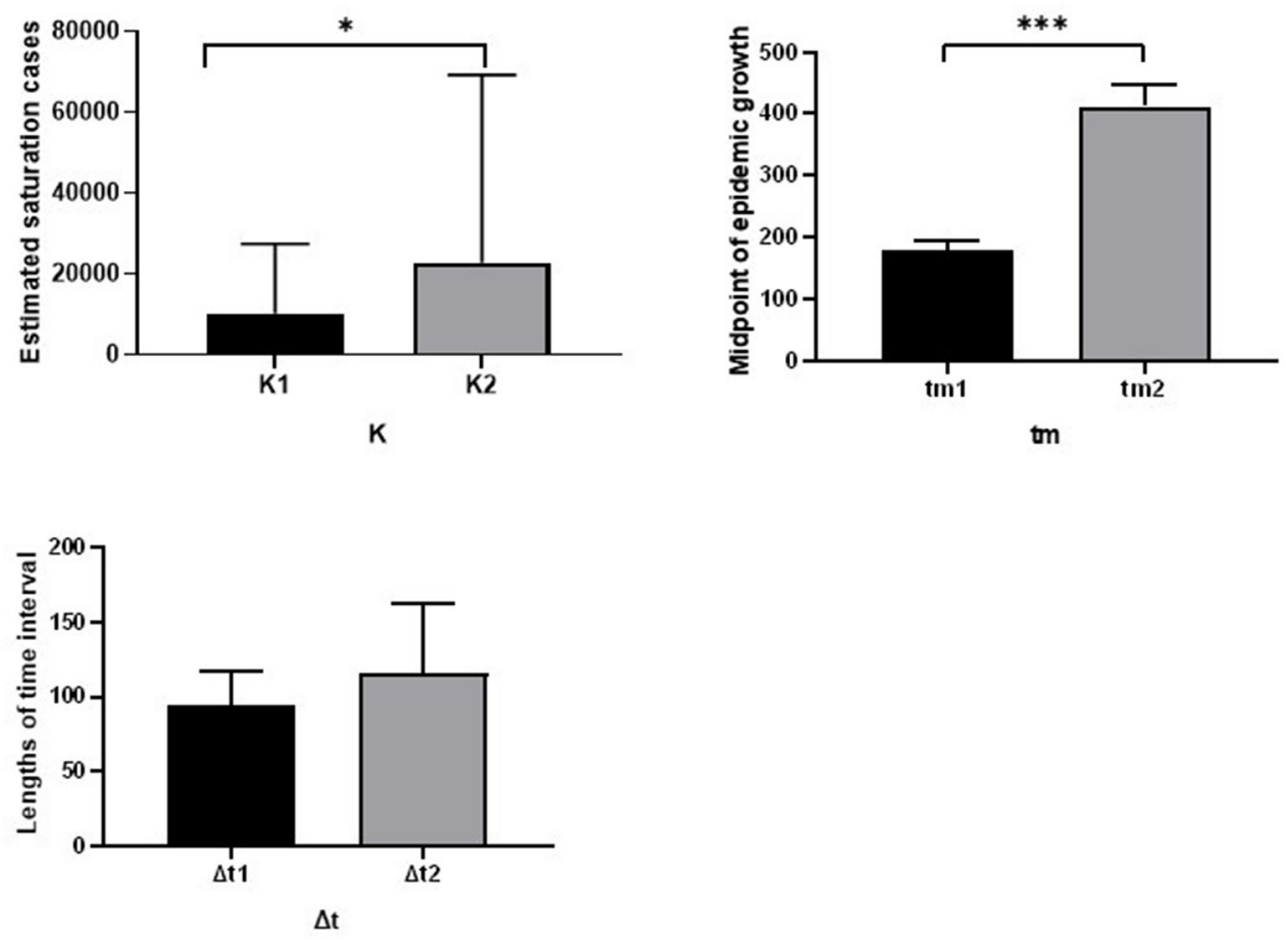
Comparison of the fitted parameters for the bi-logistic approximation of 10 regions and 2 administrative cities of Ethiopia. * indicate *P* < 0.05, *** indicate *P* < 0.001.

In addition to early-stage of epidemic characteristics, this study used a bi-logistic model to investigate the characteristics of subsequent waves in 10 regions and two administrative cities. According to this investigation, all regions and administrative cities experienced two waves of COVID-19 growth, as described in Figure 2. This model estimated the saturation level of the cumulative number of confirmed COVID-19 cases (K) in Ethiopia in both waves. The average saturation level of the second outbreak was estimated to reach a saturation level of 22,788 cases, while the average value of the first outbreak was 10,217 cases. Also, the length of time intervals (*t*) required for the epidemics to grow from 10% to 90% of the saturation level was described. The average duration from 10 to 90% of the second outbreak epidemic growth was about 116 days, while the average duration of the first epidemic growth was about 95 days. In addition, the midpoint of each epidemic growth (t_m_) of the first outbreak was 179 days, while the average midpoint growth for the second outbreak was 412 days (Figure 3).

Among all regions and cities of Ethiopia, confirmed COVID-19 cases in Addis Ababa capital city were quite high compared with other regions and cities. As of April 30^th^, about 166,571 confirmed cases of COVID-19 were reported, which was 64.7 % of confirmed COVID-19 cases reported in Ethiopia. The bi-logistic growth model indicated that the estimated saturation cases for the cumulative number of confirmed COVID-19 cases of both waves were 60,016 and 157,064, respectively, in Addis Ababa (Table 2, Figure 2A).

**Table 2:** The fitted parameters for the bi-logistic approximation for the dynamics of the cumulative incidence in each region and city administration of Ethiopia.

**Regions**	**Phase**	**K_**1**_**	**Δt_**1**_**	**tm_**1**_**	**K_**2**_**	**Δt_**2**_**	**tm_**2**_**	**RMS**	**Mean of parameters**		
									**K**	**Δt**	**tm**
Addis Ababa	2	54,740	127	178	236,568	184	414	1,818	145,654	155.5	296
Afar	2	1,709	67.2	163	3,509	196	467	56.9	2,609	131.6	315
Amhara	2	6,728	104	191	9,686	102	424	141	8,207	103	307.5
Benishangul Gumuz	2	2,564	83.9	194	3,041	98.6	431	37.8	2,802.5	91.25	312.5
Dire Dawa	2	2,980	103	191	2,314	45.7	396	91.5	2,647	74.35	293.5
Harar	2	2,798	86.6	186	3,569	102	425	85.5	3,183.5	94.3	305.5
Oromia	2	19,129	106	193	51,717	188	445	396	35,423	147	319
Sidama	2	3,521	62.2	171	7,078	100	395	171	5,299.5	81.1	283
Somali	2	1,663	124	149	1,451	63	415	35.8	1,557	93.5	282
SNNP	2	4,304	87.6	192	8,029	103	417	117	6,166.5	95.3	304.5
Unspecified	2	8,685	142	184	41,986	225	544	501	25,335.5	183.5	364

*SNNP: Southern Nations Nationalities and People. Unspecified: refers to areas where we cannot obtain public data. It is obtained by subtracting the data of 9 regions and 2 cities which have publicly available data from the national data*.

*The parameters K1, K2, and K represent the asymptotic values that bound the function and therefore specify the level at which the epidemic saturates; tm1 and tm2 represent the midpoint of each epidemic growth and hence the peak of each outbreak; Δt1 and Δt2 are the lengths of time intervals required for the epidemics to grow from 10 to 90% of the saturation level, as defined by the bi-logarithmic function*.

### COVID-19 Epidemic Size in Various Ethiopian Regions

Epidemic size per 100,000 individuals was performed in all regions and administrative cities of Ethiopia. The epidemic size per 100,000 individuals of Addis Ababa city was 4,851, which is the highest compared with the rest of the country. This was followed by the Harari region of 1,669 per 100,000 individuals. Nationwide, the average epidemic size per 100,000 individuals was 794 with a confidence interval (CI) 159-1668 (Table 1).

### Association Between Epidemic Characteristics and Current Size

**Figure 4:**
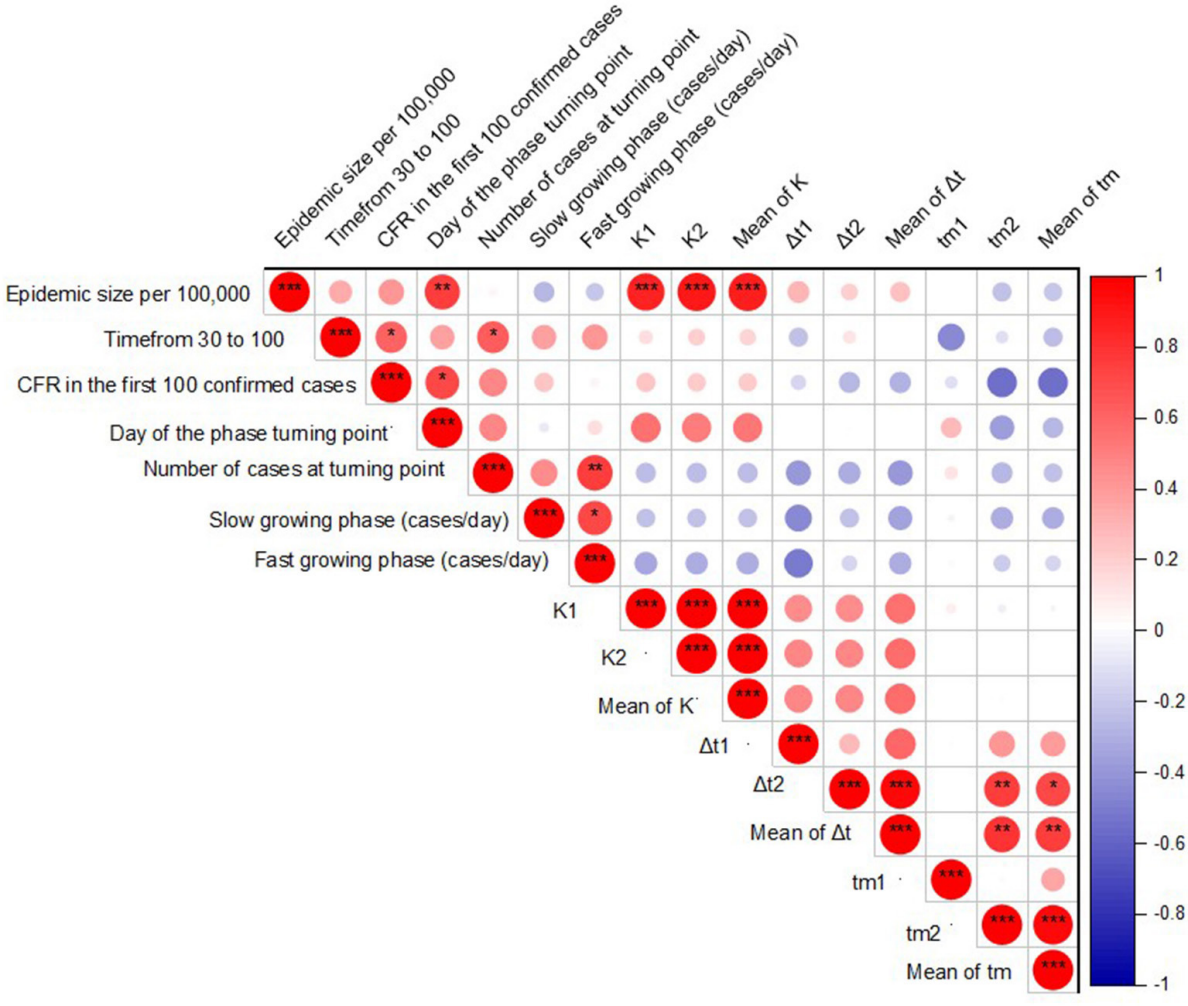
Correlation between epidemic size, early stage of epidemic indicators, and bi-logistic parameters by Spearman's correlation test. *p ≤ 0.05, **p ≤ 0.01, ***p ≤ 0.001.

We correlated the early stages of the epidemic characteristics and subsequent wave characteristics with the epidemic size as of the end of April 2021. Figure 4 indicated the correlation of epidemic size with the early stage of epidemic indicators and characteristics of subsequent waves. The epidemic size per 100,000 individuals was significantly positively correlated with the day of the phase turning point (r = 0.75, *P* = 0.008). Also, among characteristics of subsequent waves, epidemic size per 100,000 individuals was significantly positive correlated with saturation level of wave one (K_1_: r = 0.854, *P* < 0.001), wave two (K_2_: r = 0.880, *P* < 0.001), and average of saturation level (K_av_: r = 0.877, *P* < 0.001). Furthermore, bi-logistic parameters were significantly correlated with early-stage epidemic indicators. Among them, the midpoint of the second wave of epidemic growth was moderately and negatively correlated with the case fatality rate in the first 100 confirmed cases (r = 0.54). The fast-growing phase was also moderately and negatively correlated with the lengths of time intervals (Δt1) required for the epidemics to grow from 10 to 90% of the saturation level (r = 0.51).

This study also identified the differences between fitted parameters. According to this finding, the average value of the second outbreak (Mean of K_2_) is significantly greater than the average value of the first outbreak (Mean of K_1_). In addition, the midpoint growth of the second outbreak (412 days) was significantly greater than the midpoint growth of the first outbreak (179 days), as shown in Figure 3.

## Discussion

Our study has several important findings. First, we investigated the early characteristics of the epidemic in the first 100 confirmed COVID-19 cases in Ethiopia. In the early epidemic indicators, the average number of days from the slow-growing phase turning to the fast-growing phase was determined. The average number of days of slow-growing phase turning to fast-growing phase was 18.09 days. This indicates that the duration of the slow-growing phase was about 18 days, which is a long duration compared with a previous study conducted in China which was about 6 days. Further, we identified that the growth rate of the fast-growing phase (1.18 cases/day) was higher than the growth rate of the slow-growing phase (0.37 cases/day). Most of the regional states of Ethiopia transited from a slow-growing phase to a fast-growing phase at a level below thirty cases. Hence thirty cases can be indicators for the fast-growing phase. This finding is consistent with a previous study (28).

Like the fast-growing phase, the case fatality rate also indicates the early diagnosis and management of the spread of COVID-19. The fast-growing phase and high case fatality rate indicate inadequate diagnosis and prevention of epidemic spread (28). Our research found that the case fatality rate is different between ten regions and two administrative cities in Ethiopia. For example, the case fatality rate is higher in the Sidama regional state compared with Addis Ababa. However, the number of confirmed cases in Addis Ababa is higher than in Sidama. This may suggest a lack of unified early diagnosis and management between regions and cities. In addition, the differences in the case fatality rates in various regions are due to lack of public health infrastructure, geographical differences, and inadequate preventive interventions. The spreading of COVID-19 is associated with geographic location (33). This finding alert health policymakers not to generalize the case fatality rate in one region to other regions in Ethiopia. In addition, the average time required to increase from 30 to 100 cases was 22 days, which is longer than the duration reported in the previous study (28). This difference may be due to the delay in the diagnosis of the epidemic or the uncontrolled spread of COVID-19. The short duration indicates a rapid spread of COVID-19.

We used the bi-logistic model to investigate the characteristics of subsequent waves. All regions and administrative cities in Ethiopia have experienced two waves of COVID-19 growth. These findings indicate that the transmission period from the first confirmed case to 100 days (nearly 3 months and 1 week) is very short in all regions of Ethiopia. Since then, the spread of COVID-19 across the country has increased rapidly. The duration of the epidemics from 10 to 90% of the second outbreak (116 days) was higher than that of the first outbreak (96 days). However, there is no significant difference between them. There is no duration gap between both waves. This indicates that the waves are dependent on each other.

We further identified significant correlations between epidemic characteristics and epidemic size. The epidemic size was significantly correlated with the day of the epidemic turning point phase, which may reflect the potential ability of the healthcare system to react to control the spread of COVID-19. Understanding the characteristics of the early epidemic and the size of the epidemic may help to predict its impact on health. In addition, epidemic size per 100,000 individuals was positively correlated with the saturation level of both epidemic waves, suggesting the size of individual waves would predict the eventual epidemic size in the population.

Our research has identified important features of the epidemic in Ethiopia, and these findings may inform the health authorities to determine their gaps in controlling the spread of COVID-19. Therefore, to control the high spread of COVID-19, the government should formulate a new road map by considering the living conditions of Ethiopian citizens. Until enough vaccines are available for the population, Governments should provide minimum protection and safety for health care workers and patients at the health facility and national level, according to local conditions (34–36). Governments should guide the use of personal protective equipment and masks by increasing supplies.

The analysis also provides an early warning to the government of the *potential trajectory* of the COVID-19 epidemic in the coming months. As the rapid spread of COVID-19 continues, it is important to take preventive measures based on local conditions to reduce the spread of the pandemic. Therefore, we recommend the following measures that are very important to the government of Ethiopia and public health agencies to reduce the spread of the SARS-COV-2 pandemic until enough vaccine is available for all populations. First, persistent use of face masks across the country where it is impossible to keep social distancing. The government should enforce face masks use in public spaces. Currently, in Ethiopia, the mandatory wearing of masks is limited to the capital (Addis Ababa), whereas face mask use is low in the rest of the country. Numerous studies have demonstrated the protective effectiveness of face masks (25, 37–41). Second, frequent handwashing with soap or using hand sanitizer with moisturizers after every single activity. People have frequently used hand sanitizer or disinfectants in various parts of the country in the past few months. However, the adherence level of COVID-19 preventive measures was low (42). We recommend using a hand sanitizer with a moisturizer as running water is lacking in most parts of Ethiopia. Also, as a previous study reports, hand sanitizers with moisturizers have minimal allergies and irritation (43). Third, cultural values, owing to different customs, socioeconomic status, and education levels of Ethiopians, may affect social distancing (44). Ethiopians have a culture of sharing food and drinking coffee with their neighbors, which facilitate easy transmission of COVID-19 in the community. It is important to maintain social distancing as much as possible, especially in the field of public services. Finally, we recommend health professionals and public health institutions to work together to increase community awareness of the severity of COVID-19 and discover innovative ways to prevent it. Frequently health education for communities would be necessary.

This study has several limitations. First, we used publicly available data, which may contain underreported values that affect the results of the study. Second, there are differences in the reporting of COVID-19 status in various regions and cities in Ethiopia. Such differences might affect the quality of data. Third, since the control strategies implemented in various parts of Ethiopia are different from those of other countries, our research results may not be representative of other countries. Fourth, interventions such as COVID-19 testing may also impact on the epidemic size, but were not investigated in this study. Finally, the results of this study cannot be compared with findings in neighboring countries due to the lack of comparable studies in neighboring countries. We recommend further investigation to identify corresponding early characteristics and epidemic indicators for COVID-19 in these countries. This will enhance the control and prevention of COVID-19 in the region as a whole.

## Conclusions

The second wave of COVID-19 in Ethiopia is far greater, and its duration is longer than the first. Early phase turning point and case numbers in the subsequent waves predict its overall epidemic size. Understanding the characteristics of the epidemic and the epidemic size of COVID-19 in Ethiopia will inform authorities' decisions on the prevention and control of the epidemic.

## Data Availability Statement

We used publicly available data related to COVID-19, and details of the sources are included in the article.

## Author Contributions

AA and LZ: designed the study. AA, YT, and RL: performed the study and analyzed the data. AA and YT: wrote the manuscript. LZ: provided expert consultations. All authors participated in its design and coordination. All authors contributed to the article and approved the submitted version.

## Funding

This study is supported by the Bill & Melinda Gates Foundation (Grant number: INV-006104); National Natural Science Foundation of China (Grant number: 81950410639); Outstanding Young Scholars Support Program (Grant number: 3111500001); Xi'an Jiaotong University Basic Research and Profession Grant (Grant numbers: xtr022019003 and xzy032020032); Epidemiology modeling and risk assessment (Grant number: 20200344); Xi'an Jiaotong University Young Scholar Support Grant (Grant number: YX6J004).

## Conflict of Interest

The authors declare that the research was conducted in the absence of any commercial or financial relationships that could be construed as a potential conflict of interest.

## Publisher's Note

All claims expressed in this article are solely those of the authors and do not necessarily represent those of their affiliated organizations, or those of the publisher, the editors and the reviewers. Any product that may be evaluated in this article, or claim that may be made by its manufacturer, is not guaranteed or endorsed by the publisher.
